# Systematic review of the performance and clinical utility of point of care HIV-1 RNA testing for diagnosis and care

**DOI:** 10.1371/journal.pone.0218369

**Published:** 2019-06-27

**Authors:** Clara A. Agutu, Caroline J. Ngetsa, Matt A. Price, Tobias F. Rinke de Wit, Gloria Omosa-Manyonyi, Eduard J. Sanders, Susan M. Graham

**Affiliations:** 1 Kenya Medical Research Institute-Wellcome Trust Research Programme, Kilifi, Kenya; 2 International AIDS Vaccine Initiative (IAVI), Department of Medical Affairs, New York, New York, United States of America; 3 Department of Global Health, Academic Medical Centre, University of Amsterdam, Amsterdam, the Netherlands; 4 School of Medicine, College of Health Sciences, University of Nairobi, Nairobi, Kenya; 5 Centre for Tropical Medicine and Global Health, Nuffield Department of Medicine, University of Oxford, Oxford, United Kingdom; 6 Departments of Global Health, Medicine, and Epidemiology, University of Washington, Seattle, Washington, United States of America; University of Ghana College of Health Sciences, GHANA

## Abstract

**Background:**

Point of-care (POC) HIV-1 RNA tests which are accurate and easy to use with limited infrastructure are needed in resource-limited settings (RLS). We systematically reviewed evidence of POC test performance compared to laboratory-based HIV-1 RNA assays and the potential utility of these tests for diagnosis and care in RLS.

**Methods:**

Studies published up to July 2018 were identified by a search of PUBMED, EMBASE, Web of Science, CINAHL and Cochrane Central Register of Controlled Trials. Studies evaluating the use of POC HIV-1 RNA testing for early infant diagnosis (EID), acute HIV infection (AHI) diagnosis, or viral load monitoring (VL), compared to centralized testing, were included. Separate search strategies were used for each testing objective.

**Results:**

197 abstracts were screened and 34 full-text articles were assessed, of which 32 met inclusion criteria. Thirty studies evaluated performance and diagnostic accuracy of POC tests compared to standard reference tests. Two of the thirty and two additional studies with no comparative testing reported on clinical utility of POC results. Five different POC tests (Cepheid GeneXpert HIV-1 Quantitative and Qualitative assays, Alere q HIV‐1/2 Detect, SAMBA, Liat HIV Quant and Aptima HIV‐1 Quant) were used in 21 studies of VL, 11 of EID and 2 of AHI. POC tests were easy to use, had rapid turnaround times, and comparable accuracy and precision to reference technologies. Sensitivity and specificity were high for EID and AHI but lower for VL. For VL, lower sensitivity was reported for whole blood and dried blood spots compared to plasma samples. Reported error rates for Cepheid GeneXpert Qual (2.0%-5.0%), GeneXpert Quant (2.5%-17.0%) and Alere q HIV‐1/2 Detect (3.1%-11.0%) were higher than in WHO prequalification reports. Most errors resolved with retesting; however, inadequate sample volumes often precluded repeat testing. Only two studies used POC results for clinical management, one for EID and another for VL. POC EID resulted in shorter time-to-result, rapid ART initiation, and better retention in care compared to centralised testing.

**Conclusions:**

Performance of POC HIV-1 RNA tests is comparable to reference assays, and have potential to improve patient outcomes. Additional studies on implementation in limited-resources settings are needed.

## Introduction

Clinical point of care (POC) testing has evolved for situations requiring fast turnaround times and those in which a centralized lab approach faces other limitations, such as difficult or costly transportation of samples [1]. POC testing is defined as near-patient testing in a hospital, doctor’s office, clinic or home, with the advantage of providing a rapid answer[1]and thus resulting in fewer patients being lost to follow up. Testing occurs while patients are on-site and reduces the burden on patients by circumventing the need for a return visit [2]. POC testing can significantly expand access to clinical laboratory testing for rural populations by eliminating the need for sample transport, laboratory and data management infrastructure, and highly trained staff [3].

Many currently available diagnostic assays for human immunodeficiency virus type 1 (HIV-1) infection, including polymerase chain reaction (PCR), enzyme-linked immunosorbent assay (ELISA) and Western blot (WB), are limited to centralized laboratories due to requirements for infrastructure and trained personnel. These laboratory-based assays are complex, expensive and time-consuming, limiting their accessibility in developing countries where the challenge of the HIV/AIDS pandemic is most severe [4]. Although rapid antibody tests have greatly expanded access to HIV diagnosis, the inability of these tests to detect HIV-1 RNA and the poor performance of current fourth generation rapid antigen/antibody assays in many high-prevalence settings [5, 6] means that resource-limited settings face challenges in the detection of infection in infants and in patients who were recently infected. In addition, HIV-1 RNA quantitation or semi-quantitative viral load (VL) testing is needed to monitor ART [4]. POC HIV-1 RNA testing could contribute to timely HIV diagnosis and improve detection of treatment failure, resulting in improved clinical outcomes and reduced HIV transmission [4].

In 2016, two POC HIV-1 RNA assays received World Health Organization (WHO) prequalification for early infant diagnosis (EID): the Alere q HIV-1/2 Detect (Alere Technologies GmbH, Jena, Germany) and the Xpert HIV-1 Qual Assay (Cepheid AB, Solna, Sweden)[7]. Additional nucleic acid amplification testing (NAAT) assays for HIV-1 RNA detection or quantitation that have received approval include the Aptima HIV‐1 Quant Dx Assay (Hologic, Inc., San Diego, USA) in 2017, Xpert HIV‐1 Viral Load (Cepheid AB, Solna, Sweden) in 2017 [7], and the Alere q HIV-1/2 VL plasma assay (“m-PIMA HIV-1/2 VL”) (Alere Technologies GmbH, Jena, Germany) in 2019 [8]. Several additional assays of this type have active applications for WHO prequalification, including the SAMBA I & II HIV-1 Semi-Q test (Diagnostics for the Real World Ltd, San Jose, USA and Cambridge, United Kingdom) and the SAMBA I & II HIV-1 Qual Whole blood test (Diagnostics for the Real World Ltd, San Jose, USA and Cambridge, United Kingdom) [9]. Other newer assays such as the Liat HIV Quant POC VL assay (Iquum, Inc., Marlborough, MA) have not yet applied for WHO prequalification [10].

In 2018, Nash et al conducted a review of the performance of the Xpert HIV‐1 Viral Load assay, reporting high correlation between POC Xpert results and those of laboratory-based reference assays (pooled Pearson correlation 0.94; pooled Spearman correlation 0.96). Bland-Altman analyses pooled from 11 identified studies were within 0.35 log/copies ml of perfect agreement [11]. Numerous field evaluations of other POC HIV-1 RNA assays have since been conducted in different countries and settings. However, to date, there is no systematic review on the performance of all currently available POC HIV-1 RNA testing assays and assessment of their uses in HIV care.

As POC HIV-1 RNA testing has become increasingly available and has been shown to be accurate and valid for multiple clinical uses, this systematic review aimed to synthesize evidence on the performance and clinical utility of POC quantitative (i.e., continuous) or qualitative (i.e., dichotomous) HIV-1 RNA testing assays for different purposes, and to identify barriers and facilitators to their scale up in resource-limited settings.

## Methods

### Eligibility criteria

Studies evaluating the use of POC HIV-1 RNA testing for EID, acute HIV infection (AHI) diagnosis, or VL monitoring, as well as studies comparing the POC HIV-1 RNA testing to centralized HIV-1 RNA testing were included. We excluded studies testing commercially prepared sample panels [12]. Publications on laboratory-based HIV-1 RNA diagnostics, POC HIV-2 RNA assays, rapid antibody HIV tests, combined rapid antibody/antigen tests, and POC assays used for diagnosis of infectious diseases other than HIV were also excluded.

### Search strategy

The search was carried out in March 2017 and updated in July 2018. PubMed, EMBASE, Web of Science, CINAHL and the Cochrane controlled trial register were searched using the following terms: (“point of care HIV-1 viral load” OR “point-of-care HIV-1 viral load” OR “Xpert HIV-1” OR “GeneXpert” OR Alere OR SAMBA) in combination with (EID OR “early infant diagnosis” OR “infant HIV infection” OR MTCT OR “mother to child transmission”) or (“ART monitoring” OR “HIV antiretroviral therapy monitoring “OR “viral load monitoring “OR “HIV-1 viral load monitoring” OR “treatment failure”) or (“acute HIV diagnosis” OR “early HIV diagnosis”). References of included studies were searched for additional relevant literature. Conference abstracts were excluded due to insufficient detail.

### Data extraction

Study titles and abstracts were checked for eligibility according to the inclusion criteria detailed above. Full-text articles were retrieved for potentially eligible studies, and the final set of included studies was agreed upon by all researchers. Data were abstracted by two independent reviewers (CAA and CJN) using a standard data abstraction form that recorded study characteristics, sample characteristics, POC assay evaluated, comparator assay (if any), test accuracy (correlation or concordance, sensitivity, specificity), and error rate. In addition, factors related to barriers or facilitators to the scale-up of POC assays were noted, including those related to human resources, supply chain management, and patient and provider attitudes.

### Quality assessment

The quality of the studies comparing POC to reference assays was assessed by two independent reviewers using eighteen of twenty-four criteria selected from the STARD guidelines for reporting diagnostic studies [13]; these criteria were selected based on relevance to the literature reviewed. The eighteen parameters appraised covered six main categories including the title; abstract and key words; introduction; methods (participant eligibility criteria and sampling, reference and index test methods and statistical methods for comparing measures of diagnostic accuracy); results (flow chart of participant sampling, turnaround time to test results for reference and index tests and reported estimates of diagnostic accuracy) and discussion (clinical applicability of the results). Each reviewer scored the publications, with disagreements resolved by discussion with all authors.

## Results

### Study selection

**Fig 1:**
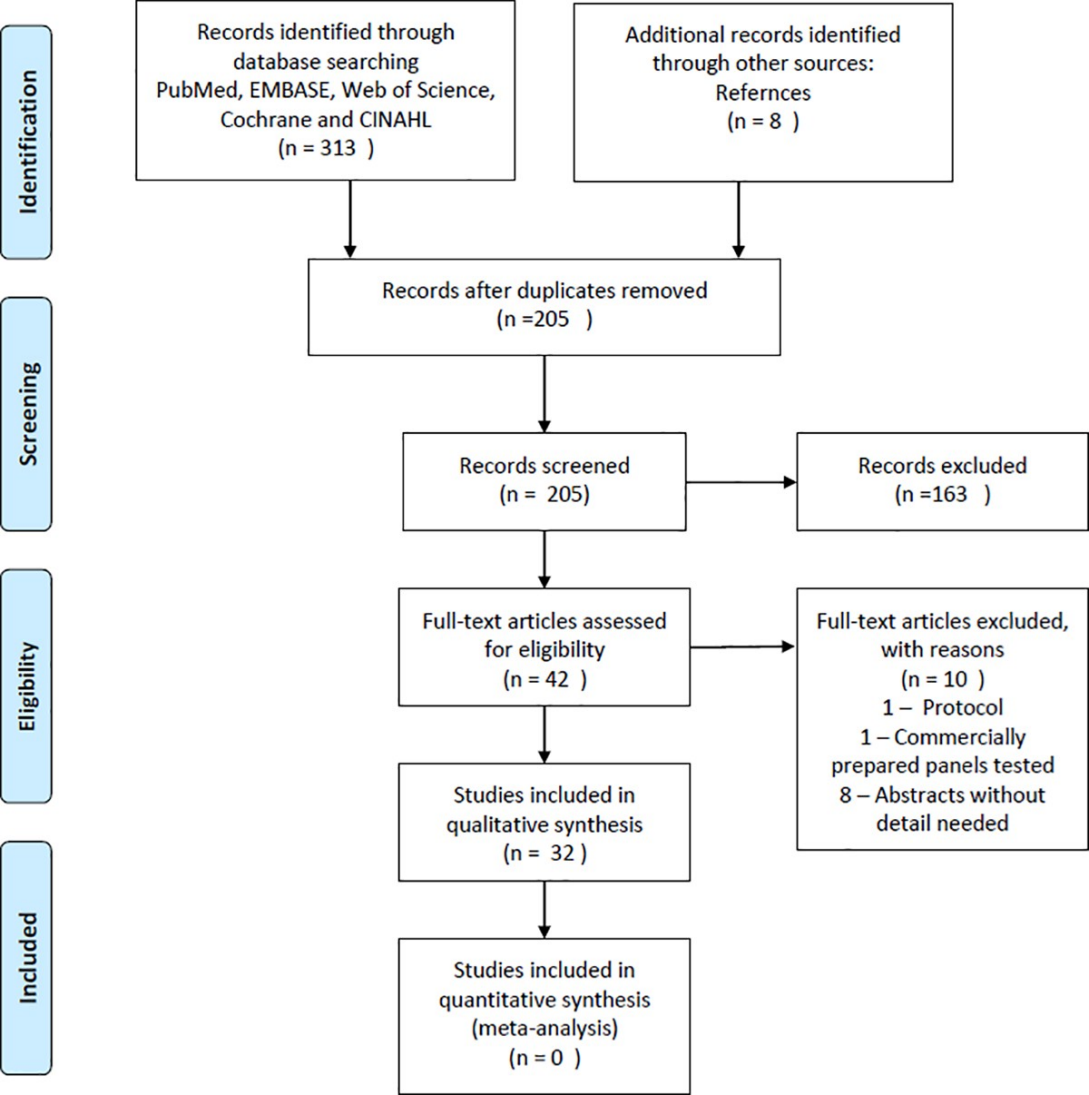
Flow diagram of study selection.

The search produced 313 references. Following removal of duplicates, 197 titles and abstracts were screened and 34 references were identified for full-text review. An additional 8 studies were identified through searches of the references of included articles. After screening was completed and any discrepancies resolved by the study team, 32 full-text articles met inclusion criteria and were included in the final review. **Fig 1** details the study selection flow chart.

### Characteristics of included studies

**Table 1** outlines the characteristics of the 32 included studies. All studies were published between 2014 and 2018. Thirty studies were field reviews comparing the performance of POC HIV-1 RNA tests to a reference standard. Two of the thirty and two additional studies with no comparative testing included reported on the clinical utility of POC results [14–17]. Of the two field reviews that reported on clinical utility [15, 17], one was a field feasibility evaluation study [14] and the other a cluster-randomized trial [16]. POC results were used for clinical management in two studies [14, 15]. The studies enrolled 12,535 infants for EID, 846 adults for AHI screening, 222 adults for HIV detection and 6,975 HIV patients for VL monitoring, and were conducted in sub-Saharan Africa (n = 16 studies, N = 14,744 participants), Europe (n = 5, N = 811), India (n = 3, N = 561), Israel (n = 4, N = 1,633), or multiple sites (n = 4, N = 2,627).

**Table 1:** Characteristics of included studies.

Clinical application	Author, year, site	POC assay	Reference assay	Sample size	Patient population	Concordance, Agreement, or Correlation	Sensitivity	Specificity	Mean difference (log copies/ml)	Error rate for POC assay
**Early infant diagnosis**	Ceffa 2016 [18] Malawi	Cepheid GeneXpert HIV-1 Qual	Abbott M2000 HIV-1 Real Time	200	HIV-exposed infants (age: ≤18 months)	Concordance: 90.9% Correlation: r = 0.95, ρ = 0.90	-	-	-	2.0%
	Dunning 2017 [19] South Africa	Alere Q HIV 1/2 Detect (Qual)	Roche CAP/CTM HIV-1 Qualitative PCR	478	HIV-exposed infants (age: <1 year)	-	100% 90.0% in infants <7 days old	100% 100% in infants <7 days old	-	9.0%
	Hsiao 2016 [20] South Africa	Alere Q HIV 1/2 Detect (Qual)	Roche CAP/CTM HIV-1 Qualitative PCR	1098	HIV-exposed children (age: <2 years)	Concordance: 99.4%	95.5% 93.3% in infants <7 days old	99.8% 100% in infants <7 days old	-	6.0% overall 10.0% in Infants <7 days old
	Ibrahim 2017 [21] Botswana	Cepheid GeneXpert HIV-1 Qual	Roche CAP/CTM HIV-1 Qualitative PCR	90	HIV-infected infants (age: <96 hours)	-	93.3%	100%	-	-
	Jani 2014 [22] Mozambique	Alere Q HIV 1/2 Detect (Qual)	Roche CAP/CTM HIV-1 Qualitative PCR	827	HIV-exposed infants (age: 1–18 months)	Concordance: 99.8% Agreement: κ = 0.98	98.5%	99.9%	-	-
	Jani 2018 [16] Mozambique	Alere Q HIV 1/2 Detect (Qual)	Roche CAP/CTM HIV-1 Qualitative PCR	3910	HIV-exposed infants (age: < 18 months)	-	-	-	-	7.0%
	Meggi 2018 [17] Mozambique	Alere Q HIV 1/2 Detect (Qual)	Roche CAP/CTM HIV-1 Qualitative PCR	2350	HIV-exposed infants (age:4 and 24 hours)	Agreement: κ = 1.00	100%	100%	-	11.0% for birth testing 3.1% in infants 4–6 weeks old
	Murray 2017 [23] South Africa	Alere Q HIV 1/2 Detect (Qual)	Roche CAP/CTM HIV-1 Qualitative PCR	322	HIV-exposed infants (age: <18 months)	Concordance: 97.8%	99.0%	99.5%	-	3.3%
		Cepheid GeneXpert HIV-1 Qual							-	2.1%
	Ndlovu 2018 [14] Zimbabwe	Cepheid GeneXpert HIV-1 Qual	-	277	HIV-exposed infants (age: 6 weeks -18 months)	-	-	-	-	4.0%
	Ondiek 2017 [24] Kenya, Uganda, Zimbabwe	SAMBA HIV-1 Qual	Roche CAP/CTM HIV-1 Qualitative PCR	745	HIV-exposed and HIV-positive infants (age not specified)	-	98.5%	99.8%	-	-
	Technau 2017 [15] South Africa	Cepheid GeneXpert HIV-1 Qual	Roche CAP/CTM HIV-1 Qualitative PCR	2238	HIV-exposed infants (age not specified)	Agreement: K = 0.967	100%	99.9%	-	5.0%
**Acute HIV infection diagnosis**	Michaeli 2016 [25] Israel	Cepheid GeneXpert HIV-1 Qual	Known true HIV-1 status by Bio-Rad Geenius HIV-1/2 confirmatory testing of follow-up samples	97	Serum samples reactive on Architect and Vidas but negative or indeterminate by Bio-Rad Geenius HIV-1/2	-	100%	92.6%-100%	-	-
	Rakovsky 2018 [26] Israel	Cepheid GeneXpert HIV-1 Qual	Known true HIV-1 status by Architect HIV Ag/Ab Combo assay and Vidas HIV DUO Ultra and indeterminate by Bio-Rad Geenius HIV-1/2	749	Serum samples reactive on Architect and Vidas 4th generation combination immunoassays but negative or indeterminate by Bio-Rad Geenius HIV-1/2 confirmatory test	-	94.9%	100%	-	-
**Acute or Chronic HIV diagnosis**	Garrett 2016 [27] South Africa	Cepheid GeneXpert HIV-1 Qual	Roche CAP CTMv2.0	20	HIV-infected adult women (median age: 33 years)	-	95%	-	-	-
	Ondiek 2017 [24] Kenya, Uganda, Zimbabwe	SAMBA HIV-1 Qual	Roche CAP/CTM HIV-1 Qualitative PCR	202	HIV-1 infected adults (age: not specified)	-	100%	99.2%	-	-
**Viral load monitoring**	Avidor 2017 [28] Israel	Cepheid GeneXpert HIV-1 Viral Load (Quant)	Roche CAP CTMv2.0	383	HIV-infected patients (age: not specified)	Correlation: r = 0.97, R^2^ = 0.94	-	-		-
	Bruzzone 2017 [29] Italy	Cepheid GeneXpert HIV-1 Viral Load (Quant)	Versant HIV-1 RNA 1.5	45	HIV-infected patients (age: not specified)	Correlation: R^2^ = 0.93	-	-	-0.13 (Xpert higher than Versant)	-
	Ceffa 2016 [18] Malawi	Cepheid GeneXpert HIV-1 Viral Load (Quant)	Abbott M2000 HIV-1 real time	300	HIV-infected children (age: ≤ 14 years) and adults (age: ≥ 15 years)	Agreement: 90.9%. Correlation: r = 0. 95, R^2^ = 0.90	-	-	0.08	8.6%
	Garrett 2016 [27] South Africa	Cepheid GeneXpert HIV-1 Viral Load (Quant)	Roche CAP CTMv2.0	42	HIV-infected adult women (median age: 33 years)	Correlation: ρ = 0.94	-	-	-0.10 (Xpert higher than Roche)	-
	Goel 2017 [30] United Kingdom, Kenya, Zimbabwe, Ukraine	SAMBA I (Semi-Quantitative)	Roche CAP CTMv2.0	520	HIV-infected adults (age: not specified)	Agreement: 98.1% at 1000 copies/ml	-	100%		-
		SAMBA II (Semi-Quantitative)	Abbott M2000 HIV-1 Real Time	150		Agreement: 98.0% at 1000 copies/ml	-	100%	-	-
	Gous 2016 [31] South Africa	Cepheid GeneXpert HIV-1 Viral Load (Quant)	Roche CAP CTMv2.0	158	HIV-infected adults (median age: = 42 years)	Concordance: 100%	92.9% at 1000 copies/ml threshold for plasma samples 60.7% for whole blood samples 50% for DBS samples	96.9% at 1000 copies/ml threshold for plasma samples 91.6% for whole blood samples 96.6% for DBS samples		2.5% for whole blood samples, 3.1% for plasma samples, 4.6% for DBS samples
			Abbott M2000 HIV-1 Real Time				100% at 1000 copies/ml threshold	95.9% at 1000 copies/ml threshold		
	Gueudin 2016 [32] France	Cepheid GeneXpert HIV-1 Viral Load (Quant)	Abbott M2000 HIV-1 Real Time	285	HIV-infected patients (age: not specified)	Correlation: ρ = 0.99	-	100%	-0.01 (Real Time higher than Xpert)	3.0%
	Hopkins 2015 [33] United Kingdom	Aptima HIV-1 Quant	Abbott M2000 HIV-1 Real Time, Qiagen Artus HI Virus-1 QS-RGQ (Artus), and Roche CAP CTMv2.0	191	HIV-infected patients (age: not specified)	Concordance: Aptima HIV-1 Quant with RealTime 95.0% at 50 copies/ml, Agreement: k = 0.74 -	-	-		-
						Concordance: Aptima HIV-1 Quant with Roche CAP CTMv2.0 88.0% at 50 copies/ml, Agreement k = 0.50 Correlation: Aptima HIV-1 Quant and the three PCR assays R^2^ > 0.93				
	Jani 2016 [34] Mozambique	Alere Q NAT (Quant)	Roche CAP CTMv2.0	443	HIV-infected adults (age; >18 years)	Correlation: r^2^ = 0.361	96.8% at 1000 copies/ml 84.0% at 10,000 copies/ml	47.8% at 1000 copies/ml 90.3% at 10,000 copies/ml		-
	Jordan 2016 [35] Europe, USA	Cepheid GeneXpert HIV-1 Viral Load (Quant)	Abbott M2000 HIV-1 real time	724	HIV-infected adults (age; ≥18 years)	Agreement: 87.2% at 40 copies/ml K = 0.63 96.6% at 200 copies/ml K = 0.93 Correlation: r = 0.98, R^2^ = 0.97	-	100%	-	3.1`%
	Kulkarni 2017 [36] India	Cepheid GeneXpert HIV-1 Viral Load (Quant)	Abbott M2000 HIV-1 real time	219	HIV-1 infected adults (mean age 37.6 years)	Concordance: 91.3% Correlation: r = 0.89, R^2^ = 0.78	97% (at 200,400 and 1000 copies/ml)	100% at 200 copies/ml 97% at 400 copies/ml, 98% at 1000 copies/ml.	0.12 (Xpert higher than Real Time)	
	Mor 2015 [37] Israel	Aptima HIV-1 Quant Cepheid GeneXpert HIV-1 Viral Load (Quant)	NucliSens v2.0 EasyQ/easyMAG assay Abbott M2000 HIV-1 real time	404	HIV-infected patients (age: not specified)	Concordance: NucliSens v2.0 vs RealTime 89.7%, vs Xpert 85.0%, vs Aptima 83.9% at 40 copies/ml Concordance: RealTime vs Xpert 89.8% vs Aptima 89.8% at 40 copies/ml Concordance: Xpert vs Aptima 91.4% at 40 copies/ml Correlation: NucliSens v2.0 vs RealTime r = 0.91, vs Xpert r = 0.90, vs Aptima r = 0.89	-	-	0.36 (NucliSens v2.0 vs Aptima)- Nuclisens lower than Aptima 0.23 (Abbott vs Aptima)–Aptima higher than Real Time 0.24 (NucliSens v2.0 vs Xpert) Nuclisens lower than Xpert 0.13 (Abbott vs Xpert)- Xpert higher than Real Time	
	Moyo 2016 [38] Botswana	Cepheid GeneXpert HIV-1 Viral Load (Quant)	Abbott M2000 HIV-1 real time	277	HIV-infected patients (age: not specified)	Agreement: 90.6% at 1000 copies/ml 97.1% at 40cp/ml Correlation: r = 0.94, r^2^ = 0.92	98.6% at 1000 copies/ml 99.6% at 40 copies/ml	-	0.34 (Xpert higher than Real Time)	
	Nash 2017 [39] India	Cepheid GeneXpert HIV-1 Viral Load (Quant)	Roche CAP CTMv2.0	246	HIV-infected adults (median age 41 years)	Correlation: r = 0.96	-	-	0.13	17.0%
	Ndlovu 2018 [14] Zimbabwe	Cepheid GeneXpert HIV-1 Viral Load (Quant	-	1302	HIV-infected adults (age; ≥18 years)	-	-	-	-	4.0%
	Ritchie 2014 [40] United Kingdom, Malawi, Uganda	SAMBA HIV Semi-Quantitative	Roche CAP CTMv2.0	488	HIV-infected adults (age; ≥18 years)	Concordance: 96.9% at 1000 copies/ml For Malawi and Uganda samples 97.8% at 1000 copies/ml For UK samples	-	100%		
	Schalasta 2016 [41] Germany	Aptima HIV-1 Quant	Roche CAP CTMv2.0 with High Pure System (HPS/CTM)	74	HIV-infected patients (age: not specified)	Agreement: 90.1% k = 0.829	-	-	0.17 (Aptima higher than HPS/CTM	
	Schonning 2017 [42] Denmark	Aptima HIV-1 Quant	Roche CAP CTMv2.0	216	Stored clinical specimens	Agreement: 86.0% at 50 copies/ml k = 0.72 93.0% at 200 copies/ml k = 0.79 Correlation: r = 0.98	-	-	0.13 (Aptima higher than Roche CAP CTMv2.0)	
	Scott 2015 [10] South Africa	Liat HIV Quant (Iquum)	Roche CAP CTMv2.0	205	HIV-infected adults	Concordance: 100% for plasma assay Correlation: P_c_ = 0.96, r^2^ = 0.99	100% (Plasma At 1000 copies/ml 100% (Whole blood) At 1000 copies/ml	88.2% (Plasma), At 1000 copies/ml 41.2% (Whole blood) At 1000 copies/ml	-	1.6%
	Swathirajan 2017 [43] India	Cepheid GeneXpert HIV-1 Viral Load (Quant)	Abbott M2000 HIV-1 real time	96	HIV-infected patients	Correlation: r = 0.81	-	-	0.27 (Xpert higher than Real Time)	-
	Titchmarsh 2015 [44] Kenya	SAMBA HIV Semi-Quantitative	Roche CAP CTMv2.0	207	HIV-1 infected patients attending routine CD4/VL monitoring	Concordance: 96.5%	-	-		

Abbreviations: ART = antiretroviral therapy, LLD = lower limit of detection, TB = tuberculosis, VCT = voluntary counselling and testing.

Measures reported: r = Pearson’s correlation, ρ = Spearman’s correlation, k = Cohen’s kappa coefficient, R^2^ = coefficient of determination

Of the 32 included studies, 11 focused on EID, 2 on AHI diagnosis, and 21 on VL monitoring. Two studies assessed both EID and VL monitoring [14]^,^[18]. The studies evaluated the following POC assays: Cepheid GeneXpert Qual (5 for EID, 2 for AHI) and Cepheid GeneXpert Quantitative (Quant) assay (13 for VL), Alere q HIV‐1/2 Detect (6 for EID, 1 for VL), Aptima HIV‐1 Quant Dx Assay (4 for VL), Liat HIV Quant (1 for VL), and SAMBA I/II (1 for EID, 3 for VL). Three studies used multiple POC assays. References assays used to compare POC to laboratory-based assays are detailed in Table 1. Six studies used multiple comparator assays. In addition, the 2 studies of AHI diagnosis both used Architect HIV Ag/Ab Combo assay (AR, Abbott Diagnostics, Abbott Park, IL, USA), followed by Vidas HIV DUO ULTRA (VD, Biomérieux, Marcy-l'Etoile, France) if reactive; confirmatory testing was performed with the Geenius HIV-1/2 differentiation assay (GS, Bio-Rad Laboratories, 68 Hercules, California).

Three studies assessed the performance of POC HIV-1 RNA assays compared to laboratory-based NAAT assays for VL on specimens other than blood plasma [31, 34, 44]. One evaluated VL results using whole blood, plasma, and dry blood spots (DBS) [31]; another whole blood only [34]; and a third used leuko-depleted whole blood (i.e., filtered whole blood) [44].

### Accuracy and precision

Where correlation with quantitative reference test results was reported in the included studies, this was found to be high across all POC assays assessed: Alere q NAT [34], Cepheid GeneXpert Quant [18, 27–29, 32, 35–39, 43] and Qual assays [18], Aptima HIV‐1 Quant Dx assay [33, 37, 42], and Liat HIV Quant [10]. No information on correlation was reported for the studies performed with SAMBA HIV-1 Qual and SAMBA HIV-1 Semiquantitative tests [24, 30, 40, 44]. Most studies reported Spearman [18, 27, 32] or Pearson’s correlation coefficients (ρ and r, respectively) for quantitative HIV-1 RNA results [18, 28, 35–39, 42, 43], while seven studies reported Cohen’s Kappa coefficient (κ) for both quantitative and qualitative HIV-1 RNA results [15, 17, 22, 33, 35, 41, 42]. Percentage concordance or agreement with dichotomous reference test results (i.e., results above or below a threshold) was reported by sixteen studies [10, 18, 20, 22, 23, 30, 31, 33, 35–38, 40–42, 44], while sensitivity and specificity were reported in 17 and 18 studies respectively [10, 15, 17, 19–27, 30–32, 34–36, 40]. Eleven studies on VL monitoring reported Bland-Altman analysis results (i.e., mean difference vs. the comparator) for log_10_-transformed viral loads across the wide range of HIV-1 RNA levels [18, 27, 29, 32, 36–39, 41–43]. For all studies, an inter-assay difference of <0.5 log copies/ml, the accepted clinically relevant difference between two viral load measurements, was observed [45, 46].

### Early infant diagnosis

#### Cepheid GeneXpert HIV-1 Qual (Xpert Qual)

**Fig 2:**
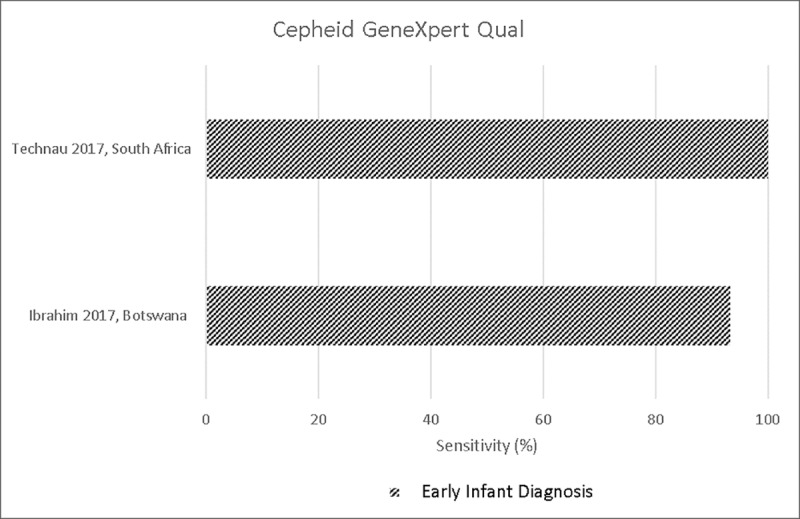
Sensitivity of Cepheid GeneXpert HIV-1 RNA Qual for early infant diagnosis.

Five studies evaluated the use of Xpert Qual for EID [14, 15, 18, 21, 23]. The Xpert Qual provides a qualitative result (HIV detectable or undetectable). A high correlation and agreement compared with reference tests was reported in two studies (r = 0.95 and k = 0.97, respectively) [15, 18]. Concordance compared to the reference test was reported by one study, and was found to be high at 90.9% [18]. Overall, sensitivity and specificity compared to reference assays ranged from 93.3%-100% and 99.5%-100%, respectively [15, 21, 23]. Xpert Qual performed well on DBS samples from infants, with 93.3% sensitivity and 100% specificity [21]. **Fig 2** presents a Forest Plot of the sensitivity of Cepheid GeneXpert Qual assay compared to reference tests for EID.

#### Alere q HIV‐1/2 Detect (Qual)

**Fig 3:**
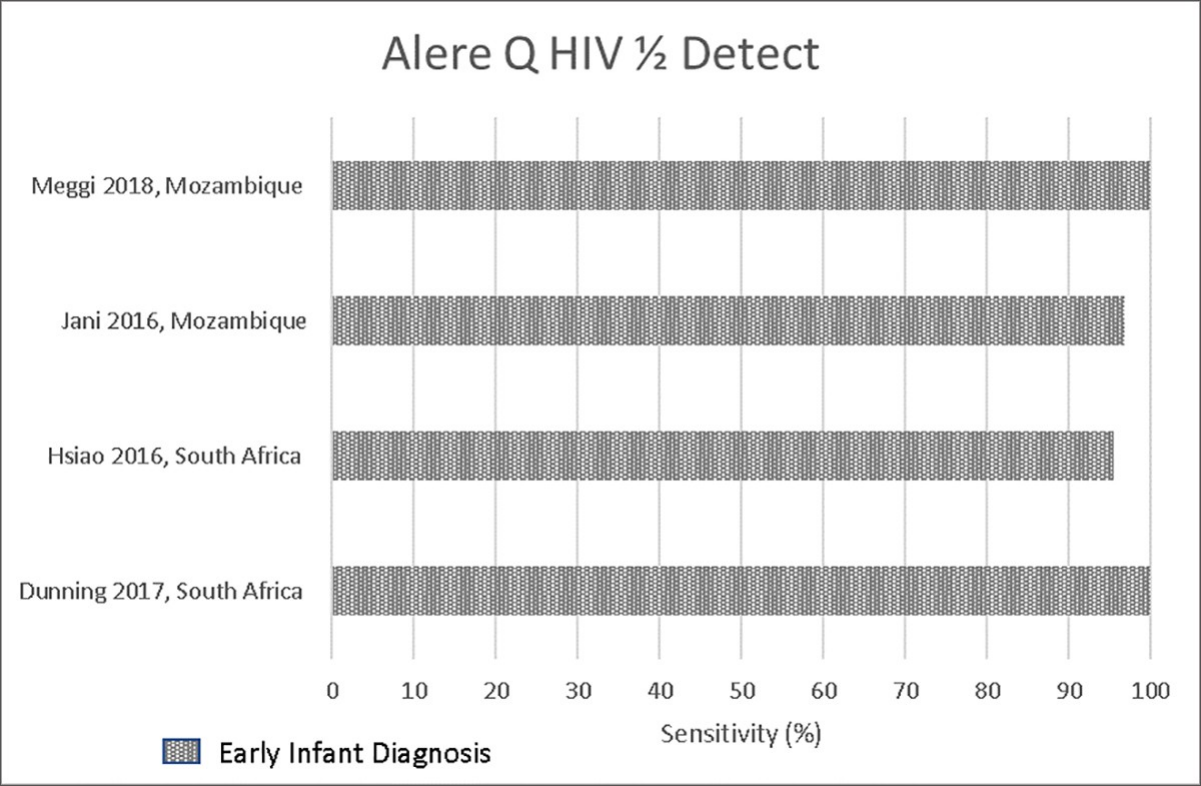
Alere Q HIV½ Detect (Qual) for Early Infant Diagnosis.

Six studies evaluated Alere Detect for EID compared to reference tests, with a qualitative result provided (HIV detected or undetected) [16, 17, 19, 20, 22, 23]. Sensitivities and specificities were high, ranging from 95.5%-100% and 99.5% -100% [17, 19, 20, 22, 23]. In two studies, sensitivity was reported to be lower in birth testing (infants <7 days old) compared to older children (routine EID at ages 6–14 weeks), at 90.0% and 93.3% compared to 100% and 95.5% respectively [19, 20]. Three studies reported high percentage agreement compared with reference tests, ranging from 97.8%-99.8% [20, 22, 23], and two studies reported on agreement by Cohen’s Kappa coefficient (κ) ranging from 0.98–1.0 [17, 22]. **Fig 3** presents a Forest Plot of the sensitivity of Alere Detect compared to reference tests for EID.

#### SAMBA HIV-1 Qual

One study assessed the performance of the SAMBA HIV-1 Qualitative test for EID compared to a reference test. High sensitivity (98.5%) and high specificity (99.8%) were reported [24].

### Acute and chronic HIV diagnosis

#### Cepheid GeneXpert HIV-1 Qual

Two studies by Michaeli et al [25] and Rakovsky et al [26] evaluated the use of Cepheid GeneXpert HIV Qual for detection of HIV-1 RNA in stored pre-seroconversion samples collected from individuals with recent, documented HIV-1 acquisition. All samples tested reactive on Architect HIV Ag/Ab Combo and Vidas HIV DUO ULTRA 4^th^ generation assays and had an indeterminate or negative result when tested by the Bio-Rad Geenius HIV-1/2 Confirmatory assay. A high sensitivity (94.9%-100%) and specificity (92.6%-100%) for the detection of HIV-1 RNA was reported in the two studies [25, 26]. Of note, Cepheid GeneXpert HIV-1 Qual failed to detect HIV-1 RNA in samples from patients with VL suppressed on antiretroviral therapy, as expected [26].

Garrett et al [27] reported on the use of the Xpert Qual for the detection of HIV-1 RNA on whole blood samples from adult known HIV positive women, likely with chronic infection. Of the twenty samples collected, 13 were from patients with detectable viral loads and 7 were virologically suppressed. All except one of the participants (95% sensitivity) with low level viremia (VL 523 copies/ml) were correctly identified by the assay.

#### SAMBA HIV-1 Qual

Ondiek [24] et al evaluated the use of SAMBA HIV-1 Qual for the detection of HIV using 202 whole blood adult samples of previously known and unknown HIV status, including ART-naïve patients. It was not specified whether participants were acutely or chronically infected with HIV. They reported a sensitivity and specificity of 100% and 99.2% respectively.

### Viral load monitoring

#### Cepheid GeneXpert HIV-1 Quant

Thirteen studies evaluated the performance of the Xpert Quant for viral load monitoring compared to reference assays [14, 18, 27–29, 31, 32, 35–39, 43]. The linear detection range of the Xpert Quant is 40−10^7^ copies/ml, with results given as a quantitative value within the analytical measurement range [47]. Correlation of HIV-1 RNA levels between Xpert Quant and reference test results was reported in eleven studies, ranging from 0.81 to 0.99 [18, 27–29, 32, 35–39, 43]. Nine studies reported a mean difference in viral load vales of -0.01 to 0.34 log copies/ml for Xpert Quant compared to reference assays [18, 27, 29, 32, 36–39, 43]. Where concordance and agreement above a threshold was reported, it was found to be high, ranging from 87.2%-100% compared to reference tests [18, 31] [35–38]. In one study, agreement was 97.1% at 40 copies/ml, but only 90.6% at 1000 copies/ml, the WHO threshold for clinical decision-making for virological failure [38]. Overall, sensitivity and specificity for HIV-1 RNA detection ranged between 84%-97% and 95.9%-100% respectively, when POC assays were compared to reference assays [31, 32, 35, 36, 38]. Sensitivity compared to reference tests at the clinically relevant threshold of 1000 copies/ml ranged from 92.9%-100% [31, 36, 38], with specificity ranging from 95.9%-98% [31, 36, 38]. Low sensitivity of 60.7% and 50.0% for the detection of HIV-1 RNA >1000 copies/ml was reported for whole blood and DBS samples respectively. Specificity was comparable to plasma samples at 91.6% and 96.6% for whole blood and DBS samples respectively [31].

#### Alere q NAT (Quant)

One study evaluated the performance of Alere q NAT compared to a reference test in whole blood samples, using several potential thresholds for treatment failure. Sensitivity for identifying treatment failure was 96.8% at 1,000 copies/ml and 84.0% at 10,000 copies/ml; however, specificity was 47.8% at 1,000 copies/ml compared to 90.3% at 10,000 copies/ml [34]. Correlation of HIV-1 RNA levels by the two methods used was r^2^ = 0.361.

#### SAMBA HIV Semiquantitative assay

High agreement was reported between the SAMBA HIV Semiquantitative assay and reference tests at a threshold of 1,000 copies/ml, ranging from 96.5%-98.1% [30, 40, 44]. Two studies reported only specificity at a threshold of 1,000 copies/ml, which was 100% in both studies [30, 40].

#### Liat HIV Quant

Scott et al evaluated the performance of Liat HIV Quant compared to reference tests for VL monitoring. A high P_c_ concordance (Pearson’s correlation x a bias correlation factor) of 0.96 with the reference test was reported. Sensitivity was 100% at a threshold of 1000 copies/ml for both plasma and whole blood assays, but specificity at this threshold was only 41.2% for whole blood, compared to 88.2% for plasma assays [10]. Downward misclassification compared to reference results at the 1000 copies/ml threshold, falsely suggesting virologic suppression, was more frequent with whole blood compared to plasma samples [10].

#### Aptima HIV‐1 Quant Dx Assay

High agreement and concordance were reported between Aptima Quant Dx assay and reference tests, ranging from 83.9%-95% [33, 37, 41, 42]. At a threshold of 50 copies/ml, agreement between the Aptima Quant Dx Assay and three reference assays (Abbott M2000 HIV-1 RealTime, Artus and Roche CAP CTMv2.0) ranged from 88.0%-95.0%. Agreement was highest with Abbott M2000 HIV-1 RealTime (k = 0.74) and lowest with Roche CAP CTMv2.0 (k = 0.50) [33]. Mor et al reported concordance at a threshold of 40 copies/ml between Aptima Quant Dx Assay, Xpert Quant, and two reference assays (NucliSENS EasyQ HIV‐1 v2.0 and Abbott M2000 HIV-1 RealTime), which ranged from 83.9%-89.8%. Concordance was highest between Aptima Quant Dx assay and Xpert Quant (91.4%) [37]. Two additional studies reported high agreement between the Aptima Quant Dx Assay and reference assays, at 30 copies/ml [41] and 50 copies/ml and 200 copies/ml (details in Table 1). No studies of Aptima Quant Dx reported sensitivity or specificity. In two studies of quantitative HIV-1 RNA levels, the Aptima Quant Dx assay yielded significantly higher results than Abbott M2000 HIV-1 RealTime [33, 37]; in another, a trend was observed for higher Aptima Quant Dx results relative to Roche CAP CTMv2.0 results [42]. In three studies of the mean difference between Aptima Quant Dx and reference test results, Aptima Quant Dx viral load values were 0.13 to 0.36 log copies/ml higher than the reference assays [37, 41, 42].

### Error rates

Error rates were reported for four POC assays; Xpert Quant, Xpert Qual, Alere Detect and Liat HIV Quant. Error rates ranged between 2.0%–5.0% for Xpert Qual [14, 15, 18, 23] and between 2.5%–17.0% for Xpert Quant [14, 18, 31, 32, 35, 39], and were associated with inadequate sample volume (“error” result) [14, 15, 18, 23], incorrect sample processing or PCR inhibition (“invalid” result) [18], insufficient data collection (“no result”) [18], mechanical or cartridge errors resulting from faulty modules requiring replacement [15], incorrect pipette supply [15], device optic errors [23], probe check failures [31], and power outages [23]. Repeat testing resolved Xpert Qual errors in two studies [15, 23], with 10% of errors persisting in another [15]. In three studies using Xpert Quant [18, 32, 35], samples for which errors occurred could not be retested due to lack of extra plasma aliquots. In the study with the highest reported invalid rate (17%) for Xpert Quant [39], the error rate was attributed primarily to cartridges that were broken during shipment or were defective. For Alere Detect, error rates ranged from 3.1%–11.0% [16, 17, 19, 20, 23], including operator errors such as no or too little sample being detected, cartridge not properly locked or misaligned, and device errors, such as a connection error between the controller and processor and failure to read the barcode [23]. Specimens once retested were reported to produce valid results[23] or resolve the majority of the errors [20]. Error rates were observed to be higher in birth testing (infants<7 days) compared to testing in older infants [17, 20]. For the Liat HIV Quant, a 1.6% error rate was reported, all due to scanning errors [10].

### Clinical utility

Four studies reported on clinical utility of POC results[14–17], and two studies used POC results for clinical management [14, 15]. Technau et al reported results of POC test implementation within an EID programme in Johannesburg, South Africa. Samples were obtained from HIV-exposed infants for POC testing with the Xpert Qual and compared to the local standard of care, which was HIV PCR testing using the Roche COBAS TaqMan HIV-1 Qualitative test (version 2·0, Roche Molecular Systems, Branchburg, NJ, USA). A positive test by either assay prompted ART initiation and confirmatory testing. A total of 30 neonates were diagnosed using the POC Xpert test, all of whom initiated ART. Time to result was reduced by POC testing (median 1 day vs. 10 days for HIV PCR), leading to more rapid ART initiation in infected neonates identified by the POC test [15].

Ndlovu et al evaluated the operational feasibility of integrated HIV VL, EID and MTB/RIF testing using the GeneXpert platform[14]. At three rural health facilities in Zimbabwe, whole blood samples were collected for HIV VL testing and DBS were collected from infants for EID. POC Xpert EID and VL testing had shorter median turnaround time for result delivery (1 day for each), compared to conventional centralised testing (17 and 26 days, respectively), substantially reducing time to ART initiation and decreasing patient loss to follow-up [14].

Jani et al measured the effect of POC EID on ART initiation rates and retention in care among HIV-positive infants in Mozambique [16]. POC EID facilitated rapid diagnosis and treatment of HIV-infected infants: 89.7% of HIV positive infants in the POC arm and 12.8% in the standard of care arm initiated ART within 60 days of sample collection. At 90 days of follow-up, 61.6% of those who initiated ART in POC arm and 42.9% in the standard of care arm were retained in care [16].

Meggi et al evaluated the feasibility, performance and diagnostic yield of rapid POC EID at birth within primary health care maternity wards in Mozambique [17]. Samples obtained from HIV-exposed infants were tested at birth and at 4–6 weeks using both POC Alere q HIV‐1/2 Detect Qual and Roche COBAS TaqMan HIV-1 Qualitative test (version 2·0). Sensitivity and specificity of POC testing compared with laboratory testing at birth were 100% (95% CI 89.4±100.0) and 100% (95% CI 99.8±100.0), respectively. Notable within the study were results of four infants who tested positive for HIV infection with laboratory-based and/or POC EID nucleic acid tests at birth but tested negative at least once during follow-up while on nevirapine (NVP) prophylaxis. For all four infants, results turned positive following NVP cessation, indicating that the diagnosis at birth was correct [17].

### Provider experiences with POC assays

#### Cepheid GeneXpert HIV-1 Qual and HIV-1 Quant

**Table 2** outlines the provider experiences with POC assays as obtained from the included studies.

**Table 2:** Provider experiences of point-of-care assays.

POC assay	Specifications	Advantages	Disadvantages
Cepheid GeneXpert HIV-1 Viral Load (Quant)	Automates the test process including RNA extraction, purification, reverse transcription and cDNA real time quantification in one fully integrated cartridge. Limit of Detection (LOD): 40 copies/mL to 10,000,000 copies/mL Specimen: plasma Turnaround time (TAT): 90 minutes	▪ Rapid TAT of results ▪ Test platform can also run assays for different pathogens (e.g., TB, hepatitis C, MRSA) ▪ Modular nature allows continuation of activities even if one module is not working. ▪ Modular nature caters to a range of test needs from high (GX 48–80 module instruments) and medium throughput (GX 4–16) to low throughput (POC Xpert Omni platform-single module). ▪ Addition of plasma to the cartridge can be performed by less skilled personnel. ▪ Absence of mechanical requirements (i.e. extraction) Ease of transport Can be used for high volume samples (80 modules) Can quantitate all HIV-1 group M, N and O subtypes. ▪ The assay has two internal quantitative controls	▪ 1,000 μl of plasma required (Quant), which could be challenging for paediatric blood draws. ▪ Careful attention to the filling line on the transfer pipette is needed to avoid sample volume adequacy errors. ▪ Need for additional infrastructure (air-conditioning units, refrigerators for sample storage, centrifuge for plasma testing). ▪ Lack of a back-up power source ▪ Insufficient samples do not allow repeat testing for errors. Repeat testing has time and cost implications. ▪ Sample addition to the cartridge is manual. ▪ Colour of the cartridges are the same for all Xpert assays (e.g. TB, HIV) ▪ Possibility of contamination of samples when the Xpert platform is used for multiple diagnostic tests at the same time e.g. EID, VL and TB sputum samples ▪ The Xpert reagent chambers contains a highly toxic chemical compound, Guanidine thiocyanate, used for extraction of DNA and RNA. The compound must be incinerated at high temperatures (≥ 850 degrees Celsius) which may not be readily available at health facilities. ▪ Risk of overloading devices in facilities with a large patient population
Cepheid GeneXpert HIV-1 Qual	Provides a total nucleic acid based test for RNA and proviral DNA in one fully integrated cartridge using whole blood and dried blood spots (DBS) for all group M HIV-1 subtypes. The assay combines automated and integrated sample preparation, nucleic acid extraction and amplification, and detection of the target sequence using real-time reverse transcription (RT-PCR) technology. Limit of detection: Whole blood 350 copies/mL, DBS 634 copies/mL Linear range: 1,000 copies/mL to 10,000,000 copies /mL for whole blood 2,500 copies/mL to 2,500,000 copies/mL for DBS. Specimens: whole blood and DBS TAT: 90 minutes	▪ Rapid TAT of results ▪ Easy to use ▪ Results easy to interpret with a detailed printout ▪ Lower blood volumes required (100 microliters whole blood) ▪ Can use DBS samples for EID ▪ Ability to be operated by non-laboratory personnel (nurses)	
SAMBA HIV Semiquantitative assay	SAMBA I: (semi-automated) automated sample preparation performed with the SAMBAprep instrument and both amplification and detection of the target nucleic acid are performed with the semi-automated SAMBAamp instrument which requires five simple manual steps including reading of the visual results (visual detection of nucleic acid with a read out similar to that of an HIV antibody test). SAMBA II: (fully automated) Sample preparation, amplification, and detection as well as reading and interpretation of the result are fully automated. Cut off of 1000 copies/mL (Semi-quantitative) Specimen: Plasma TAT: 90–120 minutes	▪ Visual detection of results ▪ Reagents stable for up to 1 month at 55 degrees Celsius, and up to 9 months at 2–37 degrees Celsius. ▪ No cold-chain transport. ▪ Does not require a desktop or computer. ▪ Requires 4h of training. ▪ Use of whole blood from a finger or heel prick does not require skilled phlebotomists or centrifugation equipment (for the new version of SAMBA Semi-q designed for performance on SAMBA II with whole blood specimens) ▪ Detects all known HIV-1 subtypes	▪ Does not provide a specific number for the viral load (SAMBA HIV Semi-quantitative). ▪ Requirement for plasma. Weak test lines have increased risk of misinterpretation by the user.
SAMBA HIV-1 Qual whole blood test	Designed for qualitative detection of both HIV-1 proviral DNA and RNA in whole blood with results provided via a visual readout on a dipstick. Performed on the semi-automated SAMBA I system, consisting of SAMBAprep and SAMBAamp systems TAT: 120 minutes. Limit of detection: 400 copies/mL Specimens: Whole blood and DBS	▪ Results read by user via a visual readout on a lateral flow test strip. ▪ Does not require a temperature controlled environment ▪ Relies on freeze-dried reagents that can be stored at room temperature	
Alere Q HIV 1/2 Detect (Qual)	Consists of a cartridge that collects 25ul of whole blood and an instrument into which the cartridge is immediately inserted. Sample preparation, reverse transcription, amplification and detection are within a cartridge. Limit of detection: 1759 copies/mL Specimen: whole blood TAT: 60 minutes.	▪ Easy to use ▪ Ability to print out test results ▪ Short TAT ▪ Back-up power system ▪ Small amount if specimen required ▪ Finger or heel prick blood can be applied directly on to the cartridge.	▪ Lack of information given when specimens abort the cycle giving rise to an error ▪ Challenges with use of the keyboard ▪ Difficulties ejecting cartridges ▪ Runs one sample at a time ▪ Difficulties using capillaries to load samples
Alere Q NAT	Consists of a cartridge that collects 25ul whole blood and an instrument into which the cartridge is inserted. Sample preparation, reverse transcription, amplification and detection are integrated within the cartridge. The technology specifically targets HIV RNA, with detection based on competitive reported monitored amplification (CMA) technology. TAT: 60 minutes.	▪ Smaller sample volume of whole blood required compared to plasma based assays. ▪ Detects HIV-1 groups M, N and O and HIV-2.	
Aptima HIV‐1 Quant Dx Assay	Based on Hologic real time transcription-mediated amplification (TMA) technology. It amplifies both the long terminal repeat (LTR) and integrase of HIV-1 on a fully automated well characterised Panther system (with random access testing). Test requires 0.7mLs and processes 0.5mLs plasma. Limit of detection: 13 copies/mL Linear detection range: 30 to 10,000,000 copies/mL Specimen: Plasma TAT: 90 minutes	▪ Fully automated, can process 320 plasma samples in 8h shift ▪ Ability to detect low copy numbers ▪ Can detect all major groups and subtypes	
Liat HIV Quant	A quantitative fully automated instrument that performs silica magnetic bead sample extraction, multiplex real time PCR amplification, and detection of HIV in a single assay tube and has a barcode reader and digital screen display with integrated keypad. It uses either 150ul plasma (Liat HIV plasma Quant assay) or 75ul whole blood (Liat HIV blood Quant assay). Limit of detection: 81 copies in 150 μl plasma, 100 to 1,500,000 copies/mL TAT 30–35 minutes.	▪ Ability to rapidly perform VL testing on both plasma and whole blood assay. ▪ Installation easy to perform and self-training within 2 hours. No supplier support needed. ▪ The analyser has a small footprint (approximately 11.4cm by 19cm by 24.1 cm) with a touch screen interface. ▪ Ease of use by non-laboratory personnel ▪ Closed system. No special safety precautions required or biohazardous waste disposal needed. ▪ Data can be exported via a USB port or Ethernet cable.	▪ The testing cartridges require cold chain (4 degrees Celsius). ▪ Plasma testing requires extra step of centrifugation ▪ Reagents have a short shelf life of 6 months.

Abbreviations: DBS: Dried Blood Spot, LOD = limit of detection, MRSA = methicillin-resistant *Staphylococcus aureus*, TAT = turnaround time, TB = tuberculosis

These assays were found to be simple to use [32, 35, 36, 43], with a rapid turnaround time of 90 minutes [18, 32, 36, 43], resulting in expeditious clinical decision-making [43]. Other advantages included: results that were easy to interpret [23], more efficient patient management compared to batch testing in a central laboratory [43], no requirement for calibrated mechanical pipettes, and the ability for less skilled personnel (potentially including non-laboratory personnel) to perform the test [15, 23, 35]. The GeneXpert platform was found to be compact in comparison to other platforms and more cost effective (≈17 USD per cartridge) [21, 36, 43]. The machine that takes a 4-cartridge module is easy to transport for use in various settings, an advantage for low-income countries [32]. The ability to run assays for tuberculosis and other pathogens in addition to HIV-1 was also advantageous [14, 18, 31, 36, 38], allowing for use of the same procurement chains and service plans for both TB and HIV diagnostics [18]. Of note, the Cepheid GeneXpert platform can be used in large laboratories with high volume, as configurations for up to 80 modules are available [31]. The modular nature of the equipment makes it possible to change one module if not working, avoiding a complete halt in the platform activities [18].

Drawbacks of the Xpert assays included the need for air-conditioning and refrigeration to maintain the platform and samples at correct temperatures [14] and the requirement for a back-up power source for the high-throughput modules when a steady power supply is not readily available [23]. In addition, overloading may occur when a large volume of tests is performed on a single machine and quality control requirements may exceed local capacity [14, 27]. Where implementation is planned for centralized testing, efficient and robust sample transport networks are needed [14]. Cartridge colours for the different Xpert assays are all the same, and therefore separate preparation areas in the laboratory are required to avoid confusion [14, 31]. Samples must also be manually added to the cartridge [23, 32]. Centrifugation is required to obtain plasma samples [14, 31, 38], and the 1000-μl plasma volume required could be a particular challenge for testing infants [38]. Finally, one study reported an instance of contamination of a DBS sample with a plasma VL sample, generating a false positive EID result and highlighting the risk of contamination with PCR assays [14].

#### Alere q HIV‐1/2 Detect Qual

Advantages of this assay include: ease of use with a short run time of about 52 minutes [23], limited training required [16, 17, 19, 22], a small sample volume requirement (25μl) [19], the ability to print out test results, and the availability of a dedicated battery pack as an alternative backup power source [23]. Disadvantages include the ability to run only one sample at a time and difficulty using capillaries to load samples [23]. The cartridge ejection mechanism was reported to cause problems and the keyboard design was not considered user-friendly. The lack of information provided when an error message occurred was reported as a further disadvantage in one study; however, valid results were obtained on re-running the specimen [19].

#### SAMBA HIV Semiquantitative assay

The short turnaround time of 90 minutes, heat stability, and limited training required have been reported as advantages [30, 40]. The visual detection of nucleic acid on a test strip was an added advantage, enabling staff to show result to the patient [40]. Limitations reported include inability to provide a quantitative result and requirement for a plasma sample, with no potential to use DBS or other sample types [30]. Despite a stronger signal on the test strip distinguishing positive results from negative results than present in current HIV rapid antibody tests, the possibility for misinterpretation of results by users remains [40, 44]. This issue can be overcome by having a paper printout of the test result, or having the results appear on a screen [40].

#### Liat HIV Quant

This assay was found easy to use, with no support required for installation and only a brief 2-hour training needed [10]. The closed system requires no special safety precautions. Data can be exported from the device using a USB port or ethernet cable. The Liat platform can test 12–14 specimens in an 8-hour day, using either whole blood or plasma. Disadvantages reported include the short half-life of reagents, the need to maintain a cold chain for the testing cartridges, and the need for centrifugation when plasma is used[10].

#### Aptima HIV‐1 Quant Dx Assay

The reported advantage of this assay is its ability to detect low HIV-1 copy numbers [33].

### Quality assessment

Quality was assessed based on the 18 modified STARD criteria for the 30 included studies that reported on diagnostic accuracy **(Table 3).** Most (86.7%, n = 26) were easily classified as studies of diagnostic accuracy, with a clear objective of comparing the diagnostic accuracy of a POC assay to one or more laboratory-based reference assays. All studies stated whether or not their results were applied clinically. Few studies, however, specified inclusion or exclusion criteria (33.3%, n = 10), clinical and demographic data for the population from which samples were obtained (36.6%, n = 11), data collection procedures (16.7%, n = 5), or training requirements for the POC assay evaluated (26.7%, n = 8).

**Table 3:** Quality assessment based on the STARD criteria.

	Titchmarsh 2015	Technau 2017	Swathirajan 2017	Scott 2015	Schonning 2017	Schalasta 2016	Ritchie 2014	Rakovsky 2018	Ondiek 2017	Nash 2017	Murray 2017	Moyo 2016	Mor 2015	Michaeli 2016	Meggi 2018
Identify as a study of diagnostic accuracy															
States research aim/question as estimating or comparing diagnostic accuracy															
Describes study population (setting, inclusion/exclusion)															
Describes participant recruitment															
Describes participant sampling/ stored sample procedures															
Describes data collection															
Describes the reference standard															
Sampling for reference or index tests															
Describes training of personnel reading index and reference test															
Describes statistical methods for comparing measures of diagnostic accuracy															
Study beginning and end dates of recruitment															
Clinical and demographic characteristics of participants															
Flow chart of participant sampling															
Reports turnaround time for test results															
Reports on how missing, indeterminate results were handled															
Reports on any events from performing the index or reference test															
Reports on estimates of diagnostic accuracy															
Discuss the clinical applicability of findings															
	Kulkarni 2017	Jordan 2016	Jani 2016	Jani 2014	Ibrahim 2017	Hsiao 2016	Hopkins 2015	Gueudin 2016	Gous 2016	Goel 2017	Garrett 2016	Dunning 2017	Ceffa 2016	Bruzzone 2017	Avidor 2017
Identify as a study of diagnostic accuracy															
States research aim/question as estimating or comparing diagnostic accuracy															
Describes study population (setting, inclusion/exclusion)															
Describes participant recruitment															
Describes participant sampling/ stored sample procedures															
Describes data collection															
Describes the reference standard															
Sampling for reference or index tests															
Describes training of personnel reading index and reference test															
Describes statistical methods for comparing measures of diagnostic accuracy															
Study beginning and end dates of recruitment															
Clinical and demographic characteristics of participants															
Flow chart of participant sampling															
Reports turnaround time for test results															
Reports on how missing, indeterminate results were handled															
Reports on any events from performing the index or reference test															
Reports on estimates of diagnostic accuracy															
Discuss the clinical applicability of findings															

Key: Grey box met assessment criteria

## Discussion

This systematic review of the literature aimed to synthesize evidence on the performance and clinical utility of POC quantitative or qualitative HIV-1 RNA testing assays for EID, AHI diagnosis and VL monitoring, and to identify barriers and facilitators to their scale-up in resource-limited settings. We found 32 studies that met inclusion criteria, of which 30 focused on diagnostic accuracy and 4 included results on clinical utility. Overall, the studies of diagnostic accuracy showed excellent performance. Where correlation between quantitative results was reported, it was high across all assays assessed; in addition, inter-assay differences reported were <0.5 log copies/ml, which is considered for clinical practice [45, 46]. Although POC VL assays tended to overestimate virologic failure compared to reference tests, which could lead to early switching, the use of central laboratory testing to confirm treatment failure could overcome this disadvantage [48]. In addition, while sensitivity for birth testing in infants <7 days of age was lower than for routine EID testing at 6–14 weeks, earlier diagnosis for those infants with positive POC results provides important advantages [14, 16]. Given the increasing body of evidence on the diagnostic accuracy of POC RNA testing, studies on clinical utility, implementation barriers and facilitators, and cost-effectiveness should be the focus of future research.

The WHO recommends HIV-1 testing of exposed infants at the earliest opportunity with an assay that detects HIV-1 DNA or RNA, by 4–6 weeks of age at the latest [49]. Without ART, about 50% of perinatally-infected infants progress to advanced disease by 8–12 weeks of age or die [50, 51]. Unfortunately, only half of all HIV-exposed infants in RLS receive an EID test within 2 months of age [49]. Challenges with current EID programs include loss to follow-up of 30%-80% of mother-infant pairs and late presentation of many infants, who miss out on the benefits of early ART initiation as well as lack of diversified testing and sample collection sites [49, 52]. In the two studies that used POC EID results, turnaround time was reduced to one day, leading to rapid ART initiation and reduced loss to follow-up [14, 15]. With the scale-up of prevention of mother to child transmission (PMTCT) programmes in sub-Saharan Africa, where all EID studies in this review were conducted, POC testing by non-specialized personnel in field settings could help decentralize services and improve infant outcomes [19]. Integration of POC HIV testing services for women and infants in other high-yield settings for paediatric HIV case-finding, including TB clinics, malnutrition clinics and inpatient wards, could lead to the identification of HIV-exposed or -infected infants missed by routine PMTCT programmes [52]. While effects of infant NVP prophylaxis on birth testing outcomes and algorithms for confirmatory testing before ART initiation still need to be addressed [17], POC testing for EID holds promise.

The WHO estimates that by mid-2016, more than 18 million HIV-infected individuals were receiving ART, with access increasing due to “test and treat” approaches [53, 54]. With continued scale-up, virologic monitoring to ensure treatment efficacy and combat HIV drug resistance is necessary [53, 54], particularly in low- and middle-income countries, where delayed treatment and poor care engagement can result from financial, human resource, and infrastructural barriers [55, 56]. Currently, only ≈20% of ART patients in low-middle income countries receive VL testing [54, 57, 58]. This low coverage has been attributed to a number of challenges, including poor sample referral systems, a lack of electronic data systems for results, and long turnaround times resulting in patient loss to follow-up [56].Improved health data systems are needed to flag those in need of VL testing and ensure fast turnaround times for prompt clinical decision-making [56, 58]. POC VL testing could meet a critical need, by ensuring same-day results for providers in rural or hard-to-reach areas where VL test access is currently limited [57, 58]. The advantages of POC VL assays may outweigh concerns about upward misclassification, especially as the current tendency is to switch too late, rather than too early [56]. Centralized confirmatory testing with standard-of-care assays for patients with suspected virological failure could address concerns about false positive results.

AHI diagnosis is a concern that has often been overlooked in RLS [59]. An estimated 10%–50% of all HIV transmission events may be attributable to AHI, a period associated with high transmission risk due to extremely high viral load and high infectivity of founder viruses [60]. Despite the importance of diagnosing AHI, there is currently no WHO recommendation on AHI diagnosis, and very few studies have considered this application of POC HIV-1 RNA assays [48]. New WHO recommendations to exclude acute or early HIV infection prior to initiating PrEP (pre-exposure prophylaxis) or PEP (post-exposure prophylaxis)[48] may provide the impetus needed to investigate the utility of POC HIV-1 RNA assays for AHI diagnosis among patients with symptoms of acute retroviral syndrome. In our review, we found two studies investigating performance of the Cepheid GeneXpert HIV-1 Qual for AHI diagnosis. Additional studies are on the horizon, including the Tambua Mapema Plus study, a proof-of-concept study evaluating the impact of an HIV-1 RNA testing intervention (Cepheid GeneXpert HIV-1 Qual) targeting young adult Kenyan patients aged 18–39 years who seek urgent care for symptoms associated with AHI (ClinicalTrials.gov Identifier: NCT03508908) [61]. Prompt diagnosis of AHI is needed to maximize the benefits of a test and treat approach [59]. If AHI diagnosis is augmented by assisted partner notification with HIV-1 RNA testing, partners with acute or prevalent HIV infection could also be identified and linked to ART if infected or PrEP if uninfected, maximizing impact.

Various factors should be considered prior to implementation and scale-up of POC HIV-1 RNA testing. Staff will require training and rigorous quality control measures should be put in place. Costs for reagents and consumables, shipment, customs charges, tax, service, and maintenance remain important considerations, which we were unable to address in the current review given the paucity of published data on this aspect of POC HIV-1 RNA tests. However, 2017 data from the Global Fund suggests the maximum price per test including consumables is $10.60 per test for the Aptima Quant Dx assay for VL, $16.80 per test for Xpert Quant for VL, $17.95 per tests for Xpert Qual for EID, up to $25 per test for Alere Q for EID, and $37.40 each for SAMBA I and II for VL and EID [62]. The cost of the POC-of-care equipment excluding service and maintenance varies greatly; from $12,280 for the GeneXpert IV-2 platform, $24,800 for SAMBA II, and $25,000 for Alere Q, up to $71,500 for the GeneXpert XVI and $72,000 for SAMBA I, and as high as $150,000 for the Hologic panther system on which the Aptima Quant Dx assay operates [62]. The primary strategy proposed to mitigate cost constraints is pooled procurement through PEPFAR or the Global Fund, with the hope that eventually higher demand will bring about competition and drive down costs [57].

Our review has a number of limitations. First, only published studies, of which the majority were field reviews, were included. Very few studies reported on clinical utility of the POC devices. Second, conference abstracts were excluded due to insufficient detail and inability to assess the quality of the study. This resulted in the exclusion of some relevant studies. Third, pricing data are often difficult to find and can change rapidly. Lastly, there was variability in the measures of accuracy, precision and agreement reported across the studies, which did not allow for pooling of data and limits its generalisability.

### Conclusion

This systematic review has identified a number of studies investigating POC HIV-1 RNA assays in RLS, of which most demonstrate acceptable clinical accuracy. Very few studies have investigated clinical utility and strategies for scale-up. As POC HIV-1 RNA assays are more widely evaluated for the uses discussed in this review, the requirement for plasma samples and thus continued need for trained phlebotomists and centrifugation of samples may remain a barrier in some RLS [57]. In addition, the need for additional resources such as air-conditioning, cold chain for reagents and a back-up power source may prove an additional challenge. If POC HIV testing is considered as part of an integrated laboratory network, strong tracking systems, good documentation, and robust sample transport and supply chain systems are needed. Moving forward, further research is needed on clinical utility, quality assurance, algorithms for confirming positive results, reliability of results for clinical decision-making, and cost-effectiveness [63]. In general, however, it is clear that POC HIV-1 RNA assays are here to stay and offer clear advantages that will help advance HIV prevention and care globally.

## Supporting information

S1 File Search strategy for systematic review. (PDF)

S2 File PRISMA checklist. (PDF)
