# Supplementary material for: Systematic review of the performance and clinical utility of point of care HIV-1 RNA testing for diagnosis and care
Source: PLoS One. 2019 Jun 27;14(6):e0218369. doi: 10.1371/journal.pone.0218369 (PMC6597060; doi:10.1371/journal.pone.0218369)
Supplement: S1 File — (PDF) [file pone.0218369.s001.pdf]

S1. Search strategy for systematic review

PUBMED (total=81)

(“point of care HIV-1 viral load” OR “point-of-care HIV-1 viral load” OR “Xpert HIV-1” OR “GeneXpert” OR Alere OR SAMBA) AND (EID OR “early infant diagnosis” OR “infant HIV infection” OR MTCT OR “mother to child transmission” OR “ART monitoring” OR “HIV antiretroviral therapy monitoring “OR “viral load monitoring “OR “HIV-1 viral load monitoring” OR “treatment failure” OR “acute HIV diagnosis” OR “early HIV diagnosis”) 81

EMBASE (total=63)

1. ("point of care HIV-1 viral load" or "point-of-care HIV-1 viral load" or "Xpert HIV-1" or "GeneXpert" or Alere or SAMBA).mp. [mp=title, abstract, heading word, drug trade name, original title, device manufacturer, drug manufacturer, device trade name, keyword, floating subheading] 2171
2. ("EID" or "early infant diagnosis" or "infant HIV infection" or MTCT or "mother to child transmission").mp. [mp=title, abstract, heading word, drug trade name, original title, device manufacturer, drug manufacturer, device trade name, keyword, floating subheading] 6880
3. 1 and 2= 27
4. ("acute HIV diagnosis" or "early HIV diagnosis").mp. [mp=title, abstract, heading word, drug trade name, original title, device manufacturer, drug manufacturer, device trade name, keyword, floating subheading] 161
5. 1 and 4= 0
6. ('ART monitoring' or 'HIV antiretroviral therapy monitoring' or 'viral load monitoring' or 'HIV-1 viral load monitoring' or 'treatment failure').mp. [mp=title, abstract, heading word, drug trade name, original title, device manufacturer, drug manufacturer, device trade name, keyword, floating subheading] 139192
7. 1 and 6= 36

WEB OF SCIENCE (total=135)

(('point of care HIV-1 viral load' OR 'point-of-care HIV-1 viral load' OR 'Xpert HIV-1' OR 'GeneXpert' OR Alere OR SAMBA)) *AND* **TOPIC:** ((EID OR 'early infant diagnosis' OR 'infant HIV infection' OR MTCT OR 'mother to child transmission')) 22

(('point of care HIV-1 viral load' OR 'point-of-care HIV-1 viral load' OR 'Xpert HIV-1' OR 'GeneXpert' OR Alere OR SAMBA)) *AND* **TOPIC:** (('ART monitoring' OR 'HIV antiretroviral therapy monitoring' OR 'viral load monitoring' OR 'HIV-1 viral load monitoring' OR 'treatment failure')) 70

TOPIC: (('point of care HIV-1 viral load' OR 'point-of-care HIV-1 viral load' OR 'Xpert HIV-1' OR 'GeneXpert' OR Alere OR SAMBA)) *AND* **TOPIC:** (('acute HIV diagnosis' OR 'early HIV diagnosis')) 43

CINAHL (total=2)

(“point of care HIV-1 viral load” OR “point-of-care HIV-1 viral load” OR “Xpert HIV-1” OR “GeneXpert” OR Alere OR SAMBA) AND (EID OR “early infant diagnosis” OR “infant HIV infection” OR MTCT OR “mother to child transmission” OR “ART monitoring” OR “HIV antiretroviral therapy monitoring “OR “viral load monitoring “OR “HIV-1 viral load monitoring” OR “treatment failure” OR “acute HIV diagnosis” OR “early HIV diagnosis”) 2

COCHRANE (total=32)

(“point of care HIV-1 viral load” OR “point-of-care HIV-1 viral load” OR “Xpert HIV-1” OR “GeneXpert” OR Alere OR SAMBA) AND (EID OR “early infant diagnosis” OR “infant HIV infection” OR MTCT OR “mother to child transmission” OR “ART monitoring” OR “HIV antiretroviral therapy monitoring “OR “viral load monitoring “OR “HIV-1 viral load monitoring” OR “treatment failure” OR “acute HIV diagnosis” OR “early HIV diagnosis”) 32
